# Development of white matter microstructure in relation to verbal and visuospatial working memory—A longitudinal study

**DOI:** 10.1371/journal.pone.0195540

**Published:** 2018-04-24

**Authors:** Stine K. Krogsrud, Anders M. Fjell, Christian K. Tamnes, Håkon Grydeland, Paulina Due-Tønnessen, Atle Bjørnerud, Cassandra Sampaio-Baptista, Jesper Andersson, Heidi Johansen-Berg, Kristine B. Walhovd

**Affiliations:** 1 Research Group for Lifespan Changes in Brain and Cognition, Department of Psychology, University of Oslo, Oslo, Norway; 2 Department of Physical Medicine and Rehabilitation, Unit of Neuropsychology, Oslo University Hospital, Oslo, Norway; 3 Department of Radiology, Rikshospitalet, Oslo University Hospital, Oslo, Norway; 4 Department of Diagnostic Physics, Rikshospitalet, Oslo University Hospital, Oslo, Norway; 5 The Oxford Centre for Functional MRI of the Brain (FMRIB), Nuffield Department of Clinical Neurosciences, University of Oxford, John Radcliffe Hospital, Oxford, United Kingdom; University of Pennsylvania, UNITED STATES

## Abstract

Working memory capacity is pivotal for a broad specter of cognitive tasks and develops throughout childhood. This must in part rely on development of neural connections and white matter microstructure maturation, but there is scarce knowledge of specific relations between this and different aspects of working memory. Diffusion tensor imaging (DTI) enables us to study development of brain white matter microstructure. In a longitudinal DTI study of 148 healthy children between 4 and 11 years scanned twice with an on average 1.6 years interval, we characterized change in fractional anisotropy (FA), mean (MD), radial (RD) and axial diffusivity (AD) in 10 major white matter tracts hypothesized to be of importance for working memory. The results showed relationships between change in several tracts and change in visuospatial working memory. Specifically, improvement in visuospatial working memory capacity was significantly associated with decreased MD, RD and AD in inferior longitudinal fasciculus (ILF), inferior fronto-occipital fasciculus (IFOF) and uncinate fasciculus (UF) in the right hemisphere, as well as forceps major (FMaj). No significant relationships were found between change in DTI metrics and change in verbal working memory capacity. These findings yield new knowledge about brain development and corresponding working memory improvements in childhood.

## Introduction

Development of working memory underlies the emergence of several abilities that are considered hallmarks of mature, higher level cognitive functions [1–3]. Working memory capacity develops throughout childhood [4, 5] along with a number of structural maturational processes in the brain [6–8]. A relationship between white matter microstructure, as derived from diffusion tensor imaging (DTI), and working memory in adolescence has been suggested by cross-sectional studies [9–15]. However, in order to establish the impact of development of these brain substrates on the development of working memory, longitudinal investigations are crucial. Of interest, a longitudinal study from the age of six years onwards demonstrated that regional fractional anisotropy (FA) predicted future visuospatial working memory capacity [16]. The relationships between white matter development in specific tracts and development of visuospatial and verbal working memory have not to our knowledge been investigated longitudinally, and this is the goal of the present study.

Behavioral studies investigating components of working memory in children have indicated that visuospatial and verbal working memory are relatively independent of one another at 5 and 8 years of age [17], and at 11 and 14 years of age [18]. Hence, they may also have different neural substrates, which may develop differently during childhood.

It is known that white matter microstructure changes rapidly in infancy [19–22], and that changes continue into early adulthood [12, 23, 24]. The development of white matter reflects a variety of microstructural features such as myelination, whereby axons get insulated and able to conduct action potentials at greater speeds and frequencies [24–26]. Longitudinal studies show widespread white matter fractional anisotropy (FA) increases, and mean diffusivity (MD) and radial diffusivity (RD) decreases through late childhood and adolescence, while the results for axial diffusivity (AD) are less consistent [8, 27–29].

The present study allows us to uncover possible relations between development of structural brain connectivity in specific tracts and visuospatial and verbal working memory capacity longitudinally. We recognize that a relatively wide set of tracts may potentially be of interest based on the multiple brain regions involved in working memory. Based on available cross-sectional studies, well-documented association tracts and major white matter bundles were selected as tracts of interest (TOIs): inferior longitudinal fasciculus (ILF), inferior fronto-occipital fasciculus (IFOF), superior longitudinal fasciculus (SLF), uncinate fasciculus (UF), forceps minor (FMin) and forceps major (FMaj) [9–13, 30, 31]. ILF is an occipito-temporal fiber bundle connecting occipital and temporal areas [32]. IFOF mediates a direct communication between occipital and frontal lobes, and also connects the frontal lobe with the posterior part of the parietal and temporal lobes [32, 33]. SLF is part of the fronto-parietal-occipital network and projects to most lateral regions of the temporal lobe with a characteristic C-shape [34, 35]. UF is part of a fronto-temporal connectivity and is connected to the inferior frontal lobe [15, 33]. While FMin has been found to overlap with genu of corpus callosum [34], FMaj connects the occipital lobes and crosses the midline via the splenium of the corpus callosum [30, 34, 36]. fMRI studies have shown that right lateralized networks are likely to underlie maintenance of visuospatial stimuli, while a left hemisphere dominance is thought to represent maintenance of verbal stimuli [37–39]. However, the added demand of manipulation of information maintained appears to require further bilateral neural recruitment of functionally related areas for both visuospatial and verbal stimuli [40, 41].

Based on previous empirical findings, we hypothesize that 1) visuospatial and verbal working memory development will be associated with longitudinal increase in FA and decrease in MD, RD and AD in all TOIs [9–13], and 2) a somewhat greater relationship may be found for verbal working memory in the left hemisphere, whereas visuospatial working memory may relate more strongly to tracts in the right hemisphere [37–39].

## Methods

### Participants

All participants were recruited from the Norwegian Mother and Child Cohort Study [42], undertaken by the Norwegian Institute of Public Health, to the current project [43], run by the Research Group for Lifespan Changes in Brain and Cognition (LCBC) at the Department of Psychology, University of Oslo, Norway. The project was approved by the Regional Committee for Medical and Health Research Ethics. Written informed consent was obtained from the parent/guardian for all participants and oral assent was given by participants at both time points.

A parent of each participant completed a structured interview to ascertain participant eligibility at both time points. Included participants were required to be fluent Norwegian speakers and have normal or corrected-to normal vision and normal hearing. Exclusion criteria were history of injury or disease known to affect central nervous system (CNS) function, including neurological or psychiatric illness, serious head trauma such as been unconscious, being under psychiatric treatment, use of psychoactive drugs known to affect CNS functioning, low birth weight (< 2500 g), and MRI contraindications. All participants also had scans included for neuroradiological evaluation, and these were examined by a neuroradiologist and required to be deemed free of significant injuries or pathological conditions at both time points.

Two hundred and ninety-six children met the inclusion criteria (see below) and underwent DTI scanning at time point 1 (tp1). Of these, 173 completed DTI scans at both time points, yielding a total of 123 dropouts to time point 2 (tp2). The main reason for drop out was the parent’s busy schedule (n = 45). Additionally, 21 children did not want to participate, 11 of the families had moved, 10 parents did not want their child to undergo magnetic resonance imaging (MRI) a second time, and 35 were not able to participate due to other circumstances. Finally, one child did not participate due to undisclosed health reasons at tp2. Of the 173 that had DTI scans at both time points, 14 participants (mean age = 5.5, SD = 1.0, 8 females) were excluded based on motion artifacts (see section *Correction for eddy currents and subject movement*): 10 of whom based on motion at tp1 and 4 based on motion at tp2.

To avoid possible effects of handedness, 11 left handed participants (8 females, mean age = 6.5, SD = 1.2) were excluded from the current study and are not included in further analysis. One hundred and forty-eight participants (82 females) had longitudinal data and were included. Participant characteristics for the final sample are provided in Table 1. At tp1 the age range was from 4.2 to 9.3 (M = 6.2, SD = 1.1), and at tp2 the age ranged from 5.8 to 11.0 (M = 7.8, SD = 1.1). Mean interval between scans was 1.6 years (SD = 0.1), ranging from 456 to 819 days. Interval between scans was not significantly correlated with age at tp1 (r = .13, p = .124), but was at tp2 (r = .25, p = .002) where age increased with increasing interval, but was not different for females and males (t = -1.52, p = .131).

**Table 1:** Participant characteristics and working memory performance.

	Mean	SD	Range
Age tp1	6.2	1.1	4.2–9.3
Age tp2	7.8	1.1	5.8–11.0
Interval years	1.6	0.1	1.3–2.2
Spatial Span Backwards tp1 ^a^	3.3	1.4	0–7
Spatial Span Backwards tp2 ^b^	4.4	1.0	2–7
Digit Span Backwards tp1^c^	2.7	1.1	0–5
Digit Span Backwards tp2^d^	3.4	0.9	2–6

Participant’s cognitive scores at time point 1 (tp1) and time point 2 (tp2). Number of participants;

^a^ n = 148,

^b^ n = 147,

^c^ n = 145,

^d^ n = 146.

### Neurocognitive assessment

Visuospatial and verbal working memory were assessed with the Wechsler Memory Scale—Third Edition (WMS-III) Spatial Span Backward and Digit Span Backward, respectively [44]. For the Spatial Span Backward participants retain information about the order and position of blocks pointed at by the examiner and points to the same blocks in the reversed order, while for the Digit Span Backward participants retain information about the order of a sequence of numbers being read out loud and repeat the same digits but in reversed order. To ensure that the included measures required manipulation of the retained information and active rehearsal of the visuospatial/verbal sequence, measuring working memory, the current study focused on backward sequences for both tests, and the number of items in the longest correctly recalled trial was used as each participant´s raw score. 148 participants performed Spatial Span Backward at tp1 (M = 3.3, SD = 1.4, range 0–7) and 147 participants at tp2 (M = 4.4, SD = 1.0, range 2–7), and 145 participants performed Digit Span Backward at tp1 (M = 2.7, SD = 1.1, range 0–5) and 146 participants at tp2 (M score = 3.4, SD = 0.9, range 2–6).

### MRI acquisition

At tp1, all children underwent a practice session in a mock scanner to get familiarized with the procedures, the small space and the sounds of the MRI-scanner. They were also shown an illustration video recorded at Oslo University Hospital with a child going through each step of the MRI session. This was also done at tp2 for the children that expressed concern related to the MRI session.

All MRI data was collected using a 12-channel head coil on a 1.5 T Siemens Avanto scanner (Siemens Medical Solutions) at Rikshospitalet, Oslo University Hospital. The same scanner, head coil and sequences were used at both time-points, though with a software upgrade from B17 to B19 for most participants at tp2 (n = 136). DTI was performed with the following parameters: repetition time (TR) = 8200 ms; echo time (TE) = 81 ms; voxel size = 2.0 mm isotropic; number of slices = 64; FOV = 128; matrix size = 128 x 128 x 64; b value = 700 s/mm; number of diffusion weighted directions = 32; number of b0 images = 5 (the first 33 participants were scanned with b0 = 1); A GeneRalized Autocalibrating Partially Parallel Acquisition (GRAPPA) factor of 2 was used. Acquisition time was 5 min 30 s.

Raw datasets were deidentified and transferred to Linux workstations for initial processing at the Neuroimaging Analysis Laboratory, LCBC, University of Oslo. Further analysis was conducted at the Oxford Centre for Functional Magnetic Resonance Imaging of the Brain (FMRIB), University of Oxford, and LCBC, University of Oslo.

### Correction for eddy currents and subject movement

All included DTI scans were corrected for eddy current-induced distortions as described elsewhere [45, 46]. In short, this procedure uses all diffusion weighted volumes to make a prediction (based on a Gaussian Process) what each volume “should look like” and then registers the observed volumes to that prediction using a rigid body model for the movements and assuming a first order eddy current-induce field. In some of these data sets there was signal drop-out. This is caused by a rotation (subject movement) coinciding exactly in time with the diffusion encoding and shows itself as multiplicative signal dropout across the entire slice that was affected by the movement. It can also be caused by pulsatile movement leading to a local rotation which will then manifest as a local dropout typically around the brain stem area. The eddy current correction method described above has been extended to also detecting these dropouts by comparing the observed slice to the predicted and deciding if the difference is large enough to make it an outlier among all such differences [47]. If a slice is determined to constitute an outlier it is removed and the prediction is recalculated without this slice and the new prediction is inserted as a replacement for the removed slice. Based on the eddy outlier report, all volumes with >10 slices of signal dropout detected by the eddy correction method were deemed bad. For participants (n = 85) with 1–6 bad volumes, we excluded the bad volumes and re-corrected for eddy current-induced distortions and subject movement. This was especially done for participants with sudden motion in the scanner. Participants exceeding 6 bad volumes (≤ 24 remaining volumes) were excluded from the study (see Participants section). Each DTI sequence was visually inspected, and rated for movement and artifacts on a scale from 1 to 4 (1: excellent, 2: minor movement/artifacts, 3: some movement/artifacts, 4: major movement/artifacts). Only participants with DTI scans rated excellent or had minor or some movement at both time points were included in further analyses. The manual quality control was in accordance with the eddy outlier report and no additional participants were excluded based on manual checking. The DTI scan was acquired after T1-scans in the scanning protocol. For the T1-weighted magnetization prepared rapid acquisition gradient echo (MP-RAGE) scans we used a parallel imaging technique (iPAT), acquiring multiple T1-scans within a short scan time (acquisition duration of 4 min 18 s.). If all T1-scans were deemed bad with major movement, or the participant did not want to continue scanning, the DTI sequence was not run. This explains the high success rate for inclusion of DTI scans in the current study.

### MRI analysis

Analysis of DTI data was carried out using Tract-Based Spatial Statistics (TBSS; [48]), part of FSL [49]. The gradient directions of each DWI volume were rotated according to the transformations applied during the eddy and motion correction steps [50]. After correction for eddy currents and subject movement, as described in the previous section, the DTI images were brain-extracted using BET [51]. Then, the FA and eigenvalue maps were computed by fitting a tensor model to the diffusion data. All participants’ FA data were then aligned into a common space using the nonlinear registration tool FNIRT in a process where every FA image was aligned to every other one [52, 53], using a b-spline representation of the registration warp field [54]. Next, the mean FA across participants and time points was created based on the participant’s FA image, from the current sample, that had the smallest amount of average warping when used as a target. The target was affine-aligned into MNI152 standard space and this target-to-MNI152 affine transform was combined with each participant’s nonlinear transform to the target. This single transform was then applied to each subject’s FA image bringing each image into standard space in one transformation. The resulting standard space FA images were then averaged and thinned to create a mean FA skeleton which represents the centres of all tracts common to the group. The threshold for the mean FA skeleton was set at 0.25 to reduce the likelihood of partial voluming in the borders between tissue classes, yielding a mask of 152284 white matter voxels. Each participant’s aligned FA data was then projected onto this skeleton by searching perpendicular from the skeleton for maximum FA values. We calculated maps of change between tp2 and tp1 (tp2—tp1), and the resulting data was fed into voxelwise cross-subject statistics. The FA-derived nonlinear warps were applied to the MD, RD, and AD change maps and values were projected onto the skeleton from the same voxels as in the FA analysis (i.e. the voxel with highest FA perpendicular to each point on the skeleton). MD was defined as the mean of all three eigenvalues (λ1 +λ2 + λ3/ 3), RD as the mean of the second and third eigenvalues (λ2 + λ3/ 2), and AD as the principal diffusion eigenvalue (λ1).

The probabilistic white matter tractography atlas (the Johns Hopkins University (JHU)) [55] provided with FSL was used to extract diffusivity tract values with a probability threshold of 5%. The relatively liberal threshold was chosen to accommodate for the skeleton voxels to intersect the correct tract appropriately [48]. DTI indices from the overlap between the FA skeleton and the following tracts were extracted: left and right inferior longitudinal fasciculus (ILF), left and right inferior fronto-occipital fasciculus (IFOF), left and right superior longitudinal fasciculus (SLF), left and right uncinate fasciculus (UF), forceps major (FMaj) and forceps minor (FMin). Longitudinal DTI changes across tracts with a larger sample are presented elsewhere [56]. Most white matter tracts included in the current study showed linear development. Non-linear trajectories were found for FA in left ILF, left IFOF, in left and right UF and forceps minor, and for MD in left UF, all showing a deceleration of change with age.

### Statistical analysis

We first tested whether significant change was observed in all measures of interest. In PASW Statistics 22 (SPSS, Chicago, IL), we ran paired t-tests for Spatial Span Backward scores and Digit Span Backward scores to test for differences in capacity between tp1 and tp2. Paired t-tests were also run for FA, MD, RD and AD in TOIs to test for differences between tp1 and tp2. In order to investigate to which extent development in the two cognitive measures was related, partial correlations between Spatial Span Backward and Digit Span Backward change scores were run, controlling for sex, age and interval. When controlling for age, this refers to age at tp1 for all analyses. Hereafter, in using the term “age”, we refer to chronological age at the time of scan tp1 and tp2, and “change” we refer to alteration between time points (tp2 –tp1). For all analyses including Spatial Span Backward and Digit Span Backward change scores, n = 147 and n = 143, respectively. Unless otherwise noted, all analyses were corrected for 10 comparisons (reflecting the 10 TOIs) using Bonferroni correction. The standard Bonferroni correction procedure assumes independence between the tests, but the change for each tract within each DTI metric are highly correlated. Therefore, the Bonferroni correction threshold was adjusted for the mean correlation (r) between tract-wise change within each DTI metric (http://www.quantitativeskills.com/sisa/calculations/bonfer.htm).

To illustrate change within individuals, spaghetti plots were created for Spatial Span Backward and Digit Span Backward scores, for FA and MD in all TOIs, and for RD and AD in specific tracts. An assumption-free nonparametric general additive mixed model (GAMM) was used to plot the data. As global fits, such as linear and quadratic models, may be affected by irrelevant factors, such as the sampled age range [57], the smoothing spline (GAMM) was fitted as a function of age to describe developmental trajectories across the studied age range. Curve fitting was performed using functions freely available through the statistical environment R, version 3.0.1 (http://www.r-project.org/).

To investigate how white matter microstructure changes relate to working memory changes, partial correlations were run between change in FA and MD in TOIs, and change in Spatial Span Backward and Digit Span Backward scores, controlling for age, sex, interval and motion at both time points. Motion was quantified by the eddy outlier report and motion at both time points were used as covariates in all analyses. The analyses were first performed for FA and MD in TOIs, based on these being the most general DTI metrics. The partial correlations were repeated with RD and AD for tracts shown to be significant for FA and/or MD and Spatial Span Backward or Digit Span Backward capacity. Further, to illustrate the significant associations between TOI white matter microstructure development and working memory development, change values were z-transformed and FA, MD, RD and AD change were plotted against Spatial Span Backward and Digit Span Backward change, using PASW Statistics 22 (SPSS, Chicago, IL). For each TOI change value, age, sex, interval and motion at both time points were regressed out, and for working memory change values, age, sex and interval were regressed out.

To quantify possible outlier values, Studentized Deleted Residuals (SDR) for Spatial Span Backward change scores and Digit Span Backward change scores predicted by age were calculated. Additionally, SDR for each TOI for FA, MD, RD and AD shown to be significantly associated with working memory were calculated.

In addition, to test for specificity in different tracts for each DTI metric when assessing the relationship between change TOIs and change in Spatial Span Backward and Digit Span Backward scores, partial correlations additionally controlling for mean change in FA, MD, RD and AD were run. Differences between the correlations between change in FA, MD, RD and AD in TOIs and change in Spatial Span Backward, and change in FA, MD, RD and AD in TOIs and Digit Span Backward scores were also tested for (please see Lee and Preacher, 2013). Additionally, to test the regional effect of change in Spatial Span Backward and Digit Span Backward on change in FA, MD, RD and AD across the white matter skeleton, voxelwise statistics were performed on change maps using “randomise” with 5000 permutations to control the family-wise error rate [58]. The GLMs were run with age, sex, motion at both time points and interval as covariates.

To make sure that the software upgrade at tp2 did not affect the results, the partial correlation between change in MD in TOIs, and change in Spatial Span Backward and Digit Span Backward scores was run with software upgrade as an additional covariate.

To test the effects of age and sex on change in working memory capacity, we ran a GLM with age, sex and interval on Spatial Span Backward and Digit Span Backward change scores separately. Additionally, to test the effects of age and sex on white matter microstructure change, the GLM was repeated for all DTI change metrics in TOIs with age, sex, interval and motion at both time points. To control for non-linear effects, the partial correlation analyses, were repeated controlling for age, sex, interval, motion at both time points and age^2^.

There was a drop out of children from tp1 to tp2. 114 participants performed Spatial Span Backward at tp1 only (M = 3.0, SD = 1.4, range 0–7) and 112 participants performed Digit Span Backward at tp1 only (M = 2.7, SD = 0.9, range 0–5). For the participants that were scanned at tp1 only, new analyses of DTI data were conducted using the same FSL tool and quality checking as described previously. 11 out of 114 participants were excluded based on manual quality checking and the eddy outlier report, leaving us with 103 participants that had DTI scans at tp1 only. To investigate potential drop out effects from tp1 to tp2, independent t-tests were ran to compare cognitive scores and mean FA, MD, RD and AD for participants tested at both time points and those only tested at tp1.

## Results

Results showed significant positive change for both Spatial Span Backward scores (t = 8.93, p = < .001) and Digit Span Backward scores (t = 3.91, p = < .001) across time. For white matter microstructure change (see Table 2), FA showed significant increase in all TOIs, MD and RD showed significant decrease in nine out of ten TOIs, and AD showed significant decrease in four TOIs, significant increase in one TOI, and no significant change in five TOIs. Spatial Span Backward and Digit Span Backward change scores were shown to correlate significantly with each other (r = .21, p = .015). Mean r’s of change between TOIs, with Bonferroni adjusted alpha levels for FA/MD/RD/AD were: r = .79, p = .031/ r = .69, p = .025/ r = .72, p = .027/ r = .65, p = .022, respectively.

**Table 2:** Change in DTI metrics of white matter microstructure.

Change (tp2 –tp1)									
		FA		MD		RD		AD	
Tracts	Hemisphere	t	p	t	p	t	p	t	p
ILF	left	**11.43**	**<.001**	**-9.00**	**<.001**	**-11.28**	**<.001**	**-3.54**	**.001**
	right	**7.03**	**<.001**	**-3.34**	**<.001**	**-4.92**	**<.001**	.58	.565
IFOF	left	**12.06**	**<.001**	**-8.82**	**<.001**	**-11.33**	**<.001**	**-3.27**	**.001**
	right	**8.03**	**<.001**	**-3.17**	**.002**	**-5.39**	**<.001**	1.44	.153
SLF	left	**10.30**	**<.001**	**-7.91**	**<.001**	**-9.76**	**<.001**	**-3.74**	**<.001**
	right	**6.01**	**<.001**	-.87	.384	-1.41	.162	**4.65**	**<.001**
UF	left	**14.69**	**<.001**	**-10.86**	**<.001**	**-13.89**	**<.001**	**-4.64**	**<.001**
	right	**11.15**	**<.001**	**-6.34**	**<.001**	**-9.23**	**<.001**	-.68	.499
FMaj		**7.10**	**<.001**	**-2.66**	**.009**	**-5.02**	**<.001**	1.85	.067
FMin		**9.79**	**<.001**	**-6.25**	**<.001**	**-8.70**	**<.001**	-1.90	.059

Paired t-tests between DTI metrics at time point 1 and time point 2. ILF = Inferior longitudinal fasciculus, IFOF = Inferior fronto-occipital fasciculus, SLF = Superior longitudinal fasciculus, UF = Uncinate fasciculus, FMaj = Forceps major and FMin = Forceps minor. Numbers in bold signify Bonferroni-corrected significance level p < .031/.025/.027/.022 for FA, MD, RD and AD, respectively.

**Fig 1:**
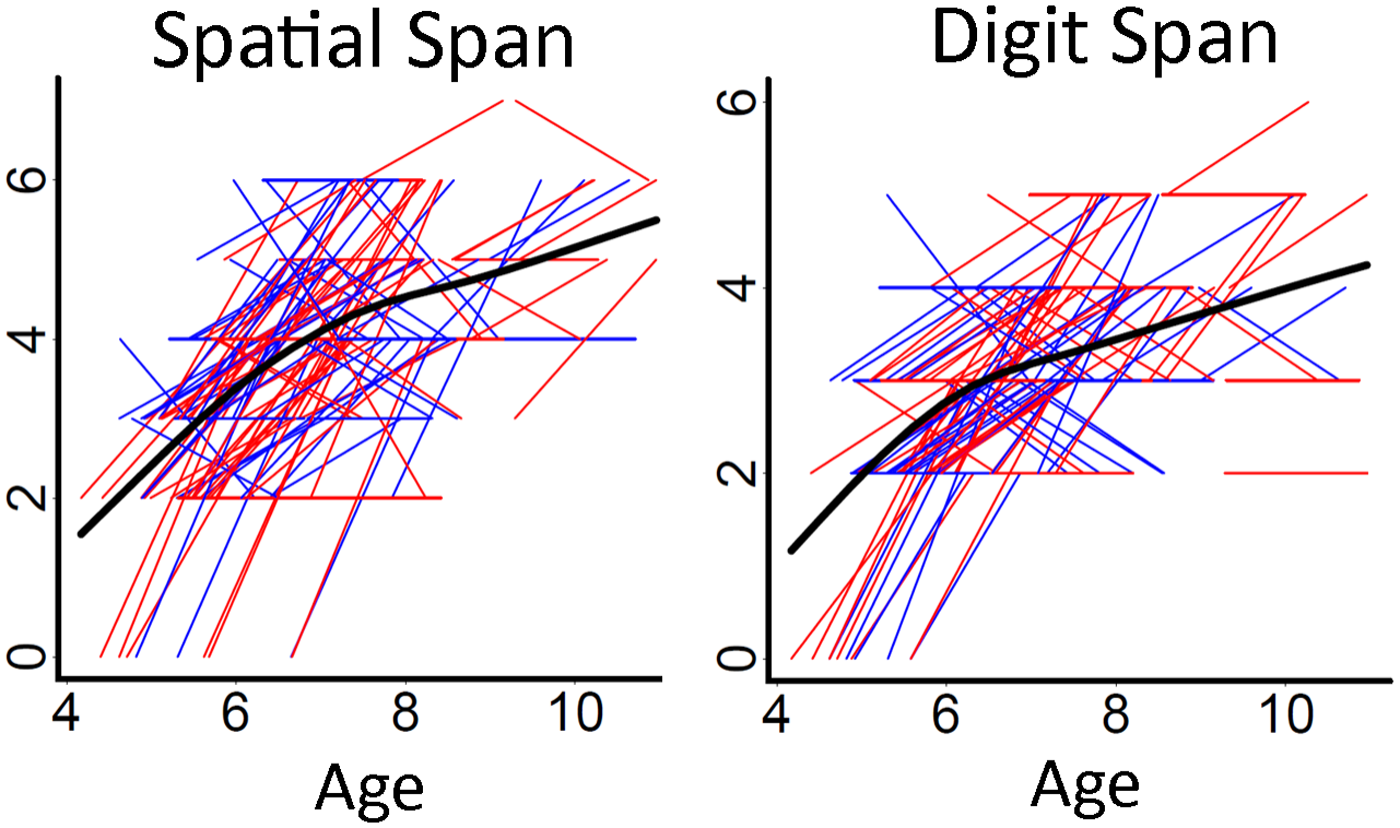
Spatial Span Backward and Digit Span Backward scores with age. Spaghetti plots of individual participant change in Spatial Span Backward and Digit Span Backward scores with age (years). Females are plotted in red and males in blue. For each measure, an assumption-free general additive mixed model as a function of age was fitted to accurately describe group-level changes across the age range.

Spaghetti plots of individual participant change from tp1 to tp2 for Spatial Span Backwards and Digit Span Backwards scores are displayed in Fig 1, where both Spatial Span Backward and Digit Span Backward showed non-linear development patterns, indicating deceleration of change with increasing age. The DTI changes across tracts with a somewhat larger sample are the topic of a separate paper [56], and the spaghetti plots of individual participant change for each white matter tract for the current sample are presented as supplementary material (please see S1 and S2 Figs).

### Change in white matter tracts and working memory change

Partial correlations showed an association between change scores for Spatial Span Backward and white matter microstructure change for FA increase in right IFOF (r = .17, p = .046), but this association did not survive multiple comparison correction (see S1 Table), and corrected significant relationships for MD decrease in four TOIs: right ILF, right IFOF, right UF and FMaj (see Table 3). Post hoc analyses were performed to test which diffusion metrics that underlie the observed effect on MD. The relationships between working memory capacity change scores and change in RD and AD in the latter four TOIs were therefore investigated (see Tables 4 and 5).

**Table 3:** MD change in white matter tracts and working memory change.

		Spatial Span Backward		Digit Span Backward	
Tract	Hemisphere	r	p	r	p
ILF	left	-.10	.235	.01	.677
	right	**-.22**	**.009**	-.01	.985
IFOF	left	-.11	.177	.04	.418
	right	**-.23**	**.007**	.02	.782
SLF	left	-.05	.526	.07	.286
	right	-.12	.146	.04	.692
UF	left	-.08	.352	.05	.233
	right	**-.20**	**.019**	-.01	.883
FMaj		**-.24**	**.004**	-.02	.977
FMin		-.15	.077	.07	.257

Partial correlation between change in MD in specific white matter tracts and change in Spatial Span Backward and Digits Span Backward scores, controlling for age, sex, interval and motion at both time points. ILF = Inferior longitudinal fasciculus, IFOF = Inferior fronto-occipital fasciculus, SLF = Superior longitudinal fasciculus, UF = Uncinate fasciculus, FMaj = Forceps major and FMin = Forceps minor. Numbers in bold signify Bonferroni-corrected significance level p < .025.

**Table 4:** RD change in white matter tracts and working memory change.

		Spatial Span Backward		Digit Span Backward	
Tract	Hemisphere	r	p	r	p
ILF	right	**-.19**	**.021**	.03	.754
IFOF	right	**-.21**	**.013**	.03	.713
UF	right	**-.19**	**.020**	.03	.771
FMaj		**-.22**	**.010**	-.01	.919

Partial correlation between change in RD in specific white matter tracts and change in Spatial Span Backward scores, controlling for age, sex, interval and motion at both time points. ILF = Inferior longitudinal fasciculus, IFOF = Inferior fronto-occipital fasciculus, UF = Uncinate fasciculus and FMaj = Forceps major. Numbers in bold signify Bonferroni-corrected significance level p < .05.

**Table 5:** AD change in white matter tracts and working memory change.

		Spatial Span Backward		Digit Span Backward	
Tract	Hemisphere	r	p	r	p
ILF	right	**-.24**	**.004**	-.05	.563
IFOF	right	**-.22**	**.008**	.01	.951
UF	right	-.16	.052	-.01	.923
FMaj		**-.24**	**.004**	.01	.923

Partial correlation between change in AD in specific white matter tracts and change in Spatial Span Backward scores, controlling for age, sex, interval and motion at both time points. ILF = Inferior longitudinal fasciculus, IFOF = Inferior fronto-occipital fasciculus, UF = Uncinate fasciculus and FMaj = Forceps major. Numbers in bold signify Bonferroni-corrected significance level p < .05.

Significant associations (p < .05) between Spatial Span Backward change and RD decrease change were found in all four TOIs: right ILF, right IFOF, right UF and FMaj, and significant associations (p < .05) between Spatial Span Backward change and AD decrease change were found in three TOIs: right ILF, right IFOF and FMaj. No significant relationships were found between change in any DTI metrics and Digit Span Backward change.

**Fig 2:**
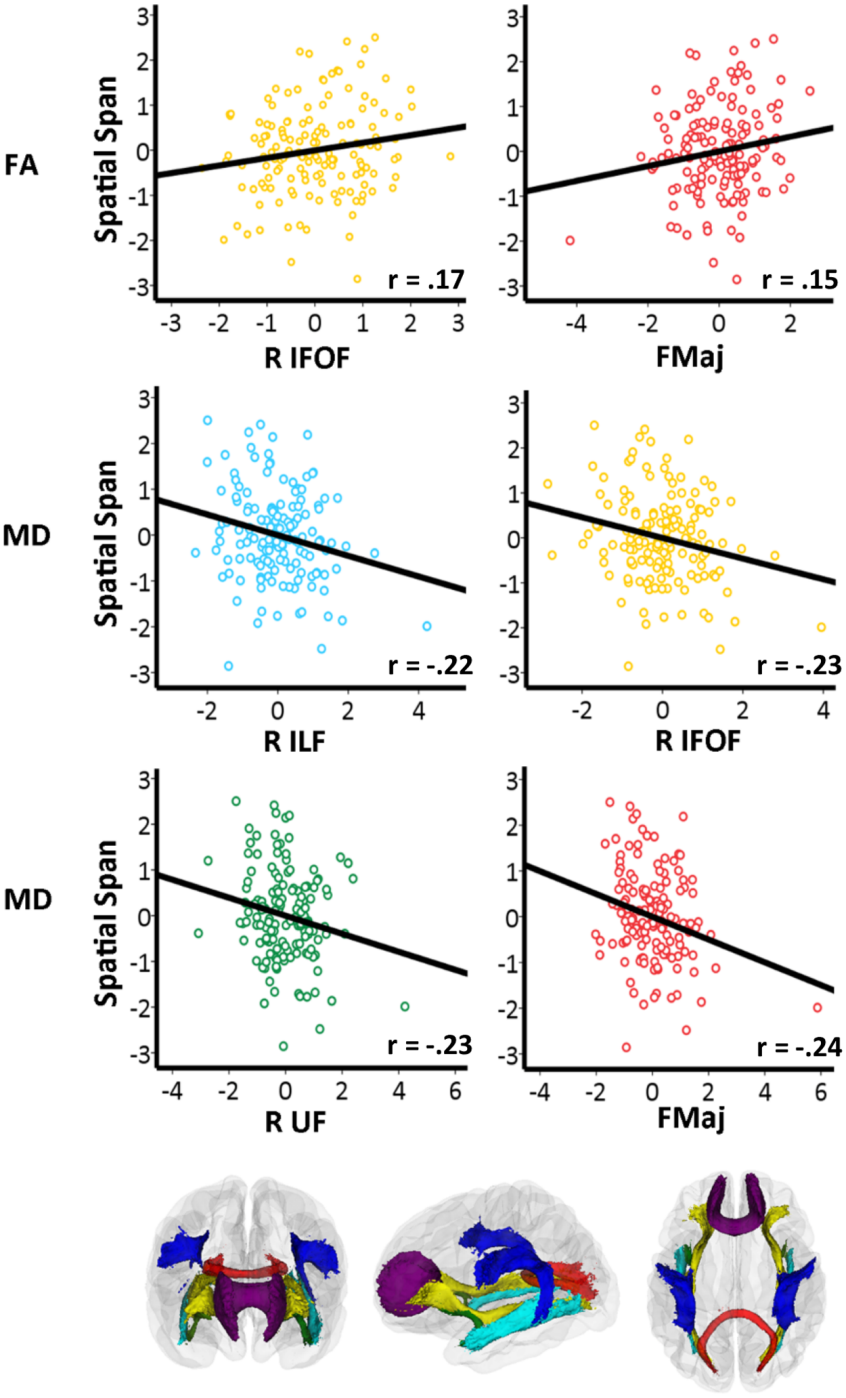
FA or MD and change in visuospatial working memory. Scatterplots showing linear relationships between change in FA or MD and change in visuospatial working memory. The plots show FA and MD in TOIs, plotted as z-transformed change values. For Spatial Span Backward scores age, sex and interval are regressed out, and for each TOI age, sex, interval and motion at both time points are regressed out. The partial correlation (r) between change in FA and MD in specific white matter tracts and change in Spatial Span Backward scores, controlling for age, sex, interval and motion at both time points are presented in each plot. The color-coded scatterplots represent the color of each specific white matter tract. Color codes refer to: Yellow: Inferior fronto-occipital fasciculus (IFOF), Red: Forceps major (FMaj), Light blue: Inferior longitudinal fasciculus (ILF) and Green: Uncinate fasciculus (UF). R = right.

**Fig 3:**
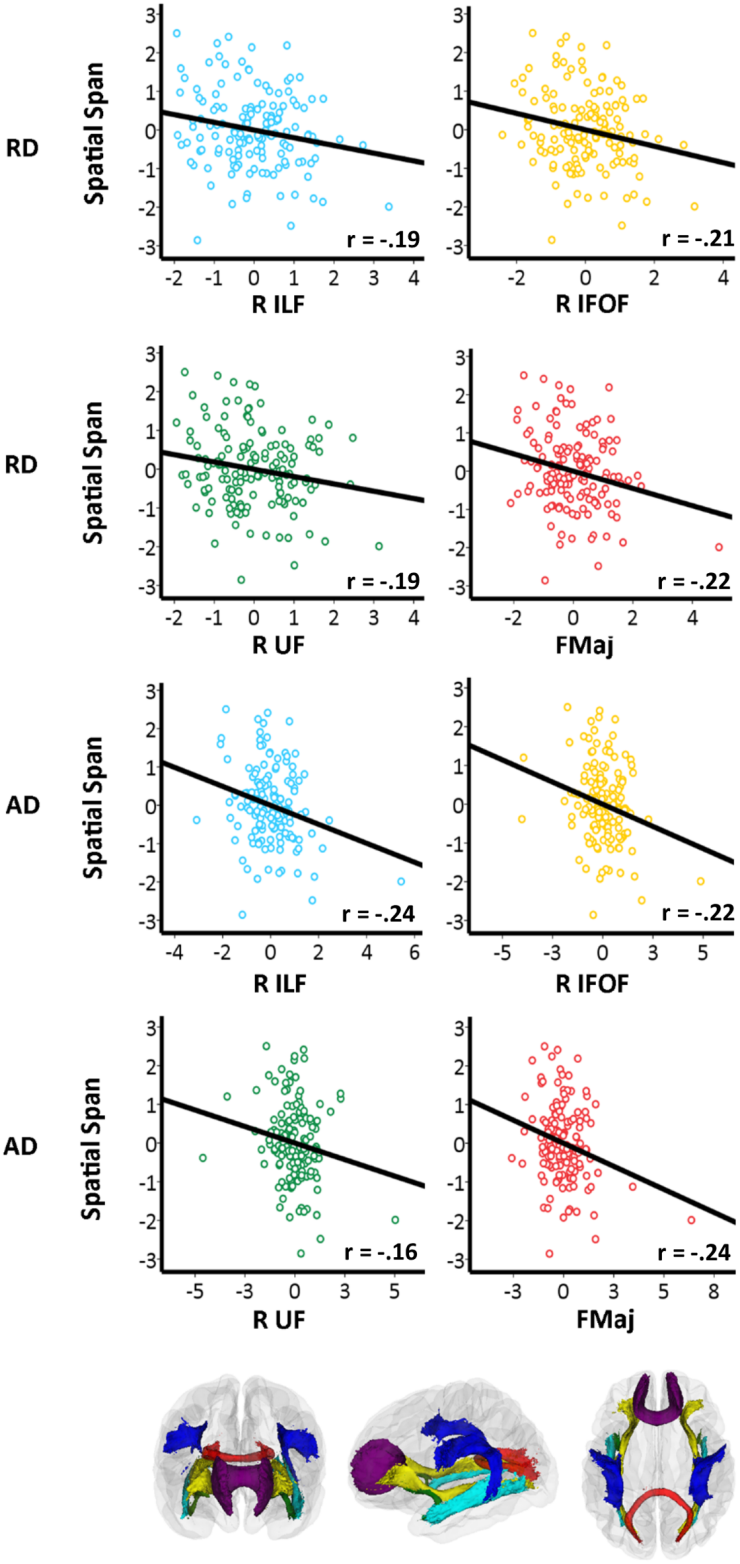
RD or AD change in visuospatial working memory. Scatterplots showing linear relationships between change in RD or AD change in visuospatial working memory in TOIs, plotted as z-transformed change values. For Spatial Span Backward scores age, sex and interval are regressed out, and for each TOI age, sex, interval and motion at both time points are regressed out. The partial correlation (r) between change in RD or AD and change in Spatial Span Backward scores, controlling for age, sex, interval and motion at both time points are presented in each plot. The color-coded scatterplots represent the color of each specific white matter tract. Color codes refer to: Light blue: Inferior longitudinal fasciculus (ILF), Yellow: Inferior fronto-occipital fasciculus (IFOF), Red: Forceps major (FMaj) and Green: Uncinate fasciculus (UF). R = right.

The main results are illustrated in Figs 2 and 3, where the relationships between change for FA (in right IFOF and FMaj) and for MD, RD and AD (in right ILF, right IFOF, right UF and FMaj) and visuospatial working memory change are shown. Overall, the plots showed increase for FA and decrease for MD, RD and AD in TOIs in relation to improving visuospatial working memory.

No participants had SDR values for Spatial Span Backward or Digit Span Backward change exceeding ±3 (SDR ranged from -2.86 to 2.93). Altogether, three participants had SDR exceeding ±3 (SDR ranged from -5.56 to 7.41) in some TOIs for FA, MD, RD or AD change. The partial correlations between change in the specific TOIs and visuospatial working memory change were repeated without these three participants to examine potential outlier effects. There were no major alterations in the results when excluding these participants (see S2 Table).

The correlations between change in FA, MD, RD and AD for TOIs and Spatial Span Backward and Digit Span Backward change scores, additionally controlling for mean change in FA, MD, RD and AD, indicates anatomic specificity for some TOIs. Meaning, a few of the correlations between TOIs and working memory were stronger compared to the correlations not controlling for mean change. This was especially found between Spatial Span Backward and FA change in right IFOF, and Spatial Span Backward and MD/RD/AD change in right ILF and right IFOF (see S3–S6 Tables).

Testing for significant differences between the correlations between TOIs and Spatial Span Backward scores, and TOIs and Digit Span Backward scores, there was significant (p < .05) difference in correlations between TOIs and Spatial Span Backward and TOIs and Digit Span Backward for; FA in right ILF (z = 1.96, p = .050), FA in right IFOF (z = 1.97, p = .050), MD in right IFOF (z = - 2.15, p = .031), AD in right IFOF (z = -1.98, p = .048), and AD in FMaj (z = - 2.16, p = .031). Voxelwise analyses showed no significant effect of change in Spatial Span Backward or Digit Span Backward on change in FA, MD, RD and AD, controlling for age, sex, motion at both time points and interval as covariates. Results from the partial correlations with software update as an additional covariate showed there was no substantial effect of software update (see S7 Table).

### Age, sex and hemisphere

GLMs testing the effect of age, showed a significant effect of age on change in Spatial Span Backward (F = 11.68, p = < .001) and Digit Span Backward (F = 6.70, p = .011), controlling for sex and interval. Significant effects of age on white matter microstructure change were found for FA, MD, RD and AD in left UF (FA: F = 5.25, p = .023/ MD: F = 7.90, p = .006/ RD: F = 7.45, p = .007/ AD: 6.28, p = .013), for FA, MD and RD in left IFOF (FA: F = 4.06, p = .046/ MD: F = 4.13, p = .044/ RD: F = 4.62, p = .033), for FA and RD in right UF (FA: F = 4.66, p = .033/ RD: F = 4.20, p = .042), and MD in FMin (F = 4.08, p = .045), controlling for sex, interval and motion. There was no significant (p < .05) effect of sex on working memory change scores, or change in FA, MD, RD and AD. Results from partial correlation analyses between change in non-linear tracts and change in working memory scores, controlling for age^2^ in addition to age showed that there was no substantial effect of adding non-linear age effects (see S8 Table).

When investigating potential drop out effects, results showed no significant difference between the two groups (tested at both time points vs tp1) for Spatial Span Backward scores (F = .044, p = .078), Digit Span Backward scores (F = 2.068, p = .821), mean FA (F = .035, p = .755), mean MD (F = .303, p = .325), mean RD (F = .283, p = .396) and mean AD (F = .083, p = .257).

## Discussion

We found significant relations between change in visuospatial working memory and change in microstructure in white matter tracts across childhood. Improvement in visuospatial working memory capacity was associated with decrease in MD in four TOIs and a tendency of increase in FA in one TOI. These relationships were driven by negative relationship between working memory change and both RD and AD change. Importantly, these relationships between fiber tract parameters and task performance did not appear to be mediated by chronological age, which was modeled as a covariate in these analyses. Specifically, improving visuospatial working memory showed significant associations with decreased MD, RD and AD in right ILF, right IFOF, right UF and FMaj, and AD in right ILF, right IFOF and FMaj. The results thus indicate that visuospatial working memory change and white matter microstructure change in part is related during childhood. No significant relationships between verbal working memory capacity change and change in DTI measures were found.

### White matter microstructure and working memory development

Cross-sectional studies have found higher FA and lower RD in IFOF to be associated with higher visuospatial working memory functioning from eight years of age to early adulthood [13]. IFOF mediates a direct communication between occipital and frontal lobes [32], suggesting a role in visuospatial working memory. In adults, object working memory has also been associated with FA in IFOF (Walsh et al., 2011). The presently observed longitudinal developmental relationship between IFOF and visuospatial working memory change fits these previous observations. Left UF has been associated with verbal working memory in tumor patients using three-dimensional fibre tracking [15]. UF plays an important role in recurrent maintenance of information, and is connected to the inferior frontal lobe [33], but has not been studied in relation to working memory in development. The current results did show relationship between right UF change and visuospatial working memory change. Nagy, Westerberg (10) found positive relationships for FA in the left SLF, left ILF and genu of corpus callosum with visuospatial working memory capacity between the age of 8 and 18, independent of the effect of age. While FMin has been found to overlap with genu of corpus callosum [34], FMaj projections are interconnected with e.g. temporal, parietal and frontal cortical areas [36] which can explain the observed associations between visuospatial working memory development and increase for FA and decrease for MD, RD and AD in FMaj in the current study [30]. The few developmental studies available in the literature exploring the relationships between white matter tract microstructure and working memory show inconclusive results with regard to regional specificity. In accordance with our finding, Østby, Tamnes (9) found no relationship between FA in SLF and verbal working memory using the same DTI measure and cognitive task as in the current study. In contrast, Peters, Szeszko (12) found positive associations between verbal working memory performance and FA in bilateral SLF, measuring white matter using a probabilistic tractography method and assessing the UMd letter-number span task. Also for visuospatial working memory, an association with higher FA and lower RD in left SLF has been found from seven years of age by using tract-based spatial statistics and assessing the Cambridge Neuropsychological Test Automated Battery SWM [11]. The same trend was found in our study, but the results were not significant. In contrast to our study, Short, Elison [59] did not find FA and RD in ILF to be associated with visuospatial working memory in 12-month-old infants, measuring white matter using deterministic fiber tracking and assessing a working memory task with infants sitting on their mothers’ laps engaging in 1–2 administrations of a 12-trial hiding game. The age difference between the latter study and our sample may explain the discrepancy in the results, as there are major changes in white matter microstructure during early childhood [60].

Most studies on the relationships between white matter tract microstructure and working memory in developmental samples have been cross-sectional, but Darki and Klingberg (16) showed in a longitudinal study that FA along the fronto-parietal and fronto-striatal white matter pathways was significantly correlated with visuospatial working memory two years later in children from six years of age. In general, cognitive performance has been associated with higher FA of white matter in cross-sectional studies, but to which extent this association is driven by maturational processes or stable characteristics is not known [61]. In the present study, we found that change over time in visuospatial working memory was associated with change in microstructural characteristics of relevant major tracts, suggesting that maturational processes and improvement in working memory seen during childhood may be related. Thus, although correlational in nature, these results go one step further in illuminating possible associations between structural brain substrates and visual working memory development. The voxelwise analysis showed no relation between changes in DTI metrics and verbal working memory development.

### Hemisphere differences

It was hypothesized that a somewhat stronger relationship would be found for verbal working memory in left hemisphere, whereas visuospatial working memory would relate more strongly to right hemisphere, in line with previous functional imaging studies [37–39].

However, we find little evidence for hemispheric differences in white matter maturation being of importance for visual vs. verbal working memory development. Also, the backward sequence for both Spatial Span and Digit Span require manipulation of the retained information, and this has been found to require bilateral neural recruitment of functionally related areas for verbal and visuospatial working memory tasks [40, 41].

### Possible neurobiological mechanisms underlying the observed relations

Within the field of neuroscience, development and maturation are highly intertwined processes. Maturation might consist of biological unfolding, physical growth and is influenced by genetics (Morishita and Hensch, 2008, Tau and Peterson, 2009, Chen et al., 2011, Chen et al., 2013). Development has been defined as the combined work of gens and environment (Berardi et al., 2015). The relevance of white matter pathways for efficient working memory performance may reflect the need for speeded and robust communication between distant brain regions. One of the underlying biological processes in white matter development is myelination, whereby axons get insulated and able to conduct action potentials at greater speeds and frequencies [24, 26]. Animal studies indicate that RD is partly related to myelination and axonal packing [25, 62]. FA and MD reflect a variety of microstructural features, including the relative alignment of individual axons, their diameter and thickness of the myelin sheath, as well as axonal density [25]. AD might reflect axonal injury and crossing fibers [63]. The relationships between increased FA and decreased MD, RD and AD in TOIs with improving working memory performance may possible be partly related to increased myelination. Still, care should be taken when considering underlying biological processes. Other processes, such as axonal alignment, axonal density and axon circumference [64] are likely also important.

### Visuospatial and verbal working memory

By investigating two different working memory functions we were able to demonstrate that development of specific white matter tracts were associated with development of visuospatial working memory, while no associations with development of verbal working memory were found. However, when tested statistically, there was no significant difference between the relation between change in white matter tracts and visuospatial working memory and verbal working memory. The weak correlation between the visuospatial and verbal working memory change measures are consistent with the hypothesis that the phonological loop and the visuospatial sketchpad components of working memory might not depend on a single storage and are somewhat independent of each other [4, 65, 66]. Behavioral studies investigating components of working memory in children have indicated that visuospatial and verbal working memory are relatively independent of one another from the age of five [17, 18]. The working memory model has been further supported by neuroimaging and neuropsychological studies that have identified distinct neuroanatomical loci for working memory systems, described in a review by Henson [67]. Still, the lack of relationship between change in verbal working memory and change in DTI metrics was not expected. Verbal working memory had a lower variability than visuospatial working memory, and this may potentially influence the results. These differences between verbal and visual working memory need to be specifically addressed in future studies.

### Effects of age

The results did show effects of age for visuospatial and verbal working memory change scores indicating more improvement in working memory capacity for the youngest participants. This was also evident from the nonparametric GAMM age functions for visuospatial and verbal working memory showing non-linear developmental trajectories. In the literature, both visuospatial and verbal working memory have shown broadly similar developmental functions, with performance increasing non-linearly from four years and leveling off around fourteen years [4, 68]. Although differences in strategy contribute to the improved performance in early childhood [69], further working memory development has been described as a quantitative change in capacity, rather than a change in strategy [68]. Taken together, the results indicate that the basic modular structure of working memory is present from four years of age, with each component undergoing sizable expansion in functional capacity throughout the early and middle school years to early adolescence.

Also for white matter microstructure, significant effects of age were found for change in FA/MD/RD/AD in left UF and for FA/MD/RD in left IFOF and right UF, and MD in FMin. These white matter tracts were also the tracts showing non-linear trajectories in the GAMM age functions. In this study, we controlled for the effect of age in all analyses. In addition, age^2^ was controlled for. However, adding non-linear age effects did not affect our main findings.

There were no significant differences in working memory scores or white matter microstructure between participants that were tested at both time points and those only tested at tp1. Because their parents accompany them to testing, the drop out may be more dependent on the guardian than the child itself. This could explain why there are no significant difference between the children that have been tested at one time point and those that were also tested at tp2.

### Limitations and future directions

Some limitations must be noted. Participants generally performed above average on tests of cognitive functioning, and may not be representative of the general population. Possible learning effects and being familiar with the test situation may also influence results. The advantage of using Spatial Span and Digit Span tasks is that we could administer the same tests to all participants ranging from 4 to 11 years of age with no ceiling effect for the older children. In the current study, the threshold for the mean FA skeleton was set at 0.25. Manual checking of the FA skeleton across all participants showed that the nonlinear alignment successfully excluded voxels that were primarily grey matter. However, there are limitations regarding partial voluming in the borders between tissue classes [48]. Further, the white matter tracts were created with a probability threshold of 5% [70]. Care must be taken though, as there is some overlap, such as for the well-documented association tracts: SLF, ILF, IFOF and UF. ILF and IFOF share most of the projections at the posterior temporal and occipital lobes, while the UF and IFOF share the projections at the frontal lobe (Wakana et al., 2004). However, all WM tracts were manually checked and deemed anatomically correct.

### Conclusions

This longitudinal study gives moderate support for the hypothesis that development of white matter microstructure in specific tracts is related to development of working memory. Relations found for visuospatial, but not verbal working memory, suggest that improvement in visuospatial working memory capacity across childhood is associated with development of white matter connections between distributed brain regions, and that the increased efficiency of those connections and the rate of cognitive development may be related.

## Supporting information

S1 Fig FA and MD in specific tracts with age. Spaghetti plots of individual participant change in FA and MD in specific tracts with age (years). Females are plotted in red and males in blue. For each measure, an assumption-free general additive mixed model as a function of age was fitted to accurately describe change across the age range. Three-dimensional renderings illustrate ten atlas-based probabilistic tracts from the Mori atlas in anterior, left, and dorsal views, displayed on a semitransparent template brain. The color-coded titles for each scatterplot represent the color of each specific white matter tract. Color codes refer to: Light blue: Inferior longitudinal fasciculus (ILF), Yellow: Inferior fronto-occipital fasciculus (IFOF), Red: Forceps major (FMaj), Blue: Superior longitudinal fasciculus (SLF), Green: Uncinate fasciculus (UF), and Purple: Forceps minor (FMin). The 3D figures were made by the use of Slicer (http://www.slicer.org/). L = left and R = right.
(TIF)

S2 Fig RD and AD in specific tracts with age. Spaghetti plots of individual participant change in RD and AD in specific tracts with age (years). Females are plotted in red and males in blue. For each measure, an assumption-free general additive mixed model as a function of age was fitted to accurately describe change across the age range. Three-dimensional renderings illustrate ten atlas-based probabilistic tracts from the Mori atlas in anterior, left, and dorsal views. The color-coded titles for each scatterplot represent the color of each specific white matter tract. Color codes refer to: Yellow: Inferior fronto-occipital fasciculus (IFOF), Light blue: Inferior longitudinal fasciculus (ILF), Green: Uncinate fasciculus (UF) and Red: Forceps major (FMaj). The 3D figures were made by the use of Slicer (http://www.slicer.org/). L = left and R = right.
(TIF)

S1 Table FA change in white matter tracts and working memory change. Partial correlation between change in FA in specific white matter tracts and change in Spatial Span Backward and Digits Span Backward scores, controlling for age, sex, interval and motion at both time points. ILF = Inferior longitudinal fasciculus, IFOF = Inferior fronto-occipital fasciculus, SLF = Superior longitudinal fasciculus, UF = Uncinate fasciculus, FMaj = Forceps major and FMin = Forceps minor.
(DOCX)

S2 Table DTI change in white matter tracts and visuospatial working memory change after excluding outliers (for each TOI). Partial correlations between change in FA, MD, RD and AD in specific white matter tracts and change in Spatial Span Backward scores, controlling for age, sex, interval and motion at both time points after excluding three participants with SDR exceeding ±3 in some TOIs for FA, MD, RD and AD predicted by age. ILF = Inferior longitudinal fasciculus, IFOF = Inferior fronto-occipital fasciculus, UF = Uncinate fasciculus and FMaj = Forceps major. Numbers in bold signify Bonferroni-corrected significance level p < .031/.025/.027/.022 for FA, MD, RD and AD, respectively.
(DOCX)

S3 Table FA change in white matter tracts and working memory change, controlling for mean FA change. Partial correlation between change in FA in specific white matter tracts and change in Spatial Span Backward and Digits Span Backward scores, controlling for age, sex, interval, motion at both time points and mean FA change. ILF = Inferior longitudinal fasciculus, IFOF = Inferior fronto-occipital fasciculus, SLF = Superior longitudinal fasciculus, UF = Uncinate fasciculus, FMaj = Forceps major and FMin = Forceps minor. Numbers in bold signify Bonferroni-corrected significance level p < .031.
(DOCX)

S4 Table MD change in white matter tracts and working memory change, controlling for mean MD change. Partial correlation between change in MD in specific white matter tracts and change in Spatial Span Backward and Digits Span Backward scores, controlling for age, sex, interval, motion at both time points and mean MD change. ILF = Inferior longitudinal fasciculus, IFOF = Inferior fronto-occipital fasciculus, SLF = Superior longitudinal fasciculus, UF = Uncinate fasciculus, FMaj = Forceps major and FMin = Forceps minor. Numbers in bold signify Bonferroni-corrected significance level p < .025.
(DOCX)

S5 Table RD change in white matter tracts and working memory change, controlling for mean RD change. Partial correlation between change in RD in specific white matter tracts and change in Spatial Span Backward scores, controlling for age, sex, interval, motion at both time points and mean RD change. ILF = Inferior longitudinal fasciculus, IFOF = Inferior fronto-occipital fasciculus, UF = Uncinate fasciculus and FMaj = Forceps major. Numbers in bold signify Bonferroni-corrected significance level p < .05.
(DOCX)

S6 Table AD change in white matter tracts and working memory change, controlling for mean AD change. Partial correlation between change in AD in specific white matter tracts and change in Spatial Span Backward scores, controlling for age, sex, interval, motion at both time points and mean AD change. ILF = Inferior longitudinal fasciculus, IFOF = Inferior fronto-occipital fasciculus, UF = Uncinate fasciculus and FMaj = Forceps major. Numbers in bold signify Bonferroni-corrected significance level p < .05.
(DOCX)

S7 Table MD change in white matter tracts and working memory change, controlling for software update. Partial correlation between change in MD in specific white matter tracts and change in Spatial Span Backward and Digits Span Backward scores, controlling for age, sex, interval, motion at both time points and software upgrade. ILF = Inferior longitudinal fasciculus, IFOF = Inferior fronto-occipital fasciculus, SLF = Superior longitudinal fasciculus, UF = Uncinate fasciculus, FMaj = Forceps major and FMin = Forceps minor. Numbers in bold signify Bonferroni-corrected significance level p < .025.
(DOCX)

S8 Table DTI change in non-linear white matter tracts and visuospatial working memory change, controlling for age^2^. Partial correlations between change in FA and MD in specific white matter tracts showing non-linear developmental patterns and change in Spatial Span Backward scores, controlling for age, sex, interval, motion at both time points and age^2^. ILF = Inferior longitudinal fasciculus, IFOF = Inferior fronto-occipital fasciculus, UF = Uncinate fasciculus and FMin = Forceps minor.
(DOCX)
