# Supplementary figures and images for: Development of white matter microstructure in relation to verbal and visuospatial working memory—A longitudinal study
Source: PLoS One. 2018 Apr 24;13(4):e0195540. doi: 10.1371/journal.pone.0195540 (PMC5916522; doi:10.1371/journal.pone.0195540)

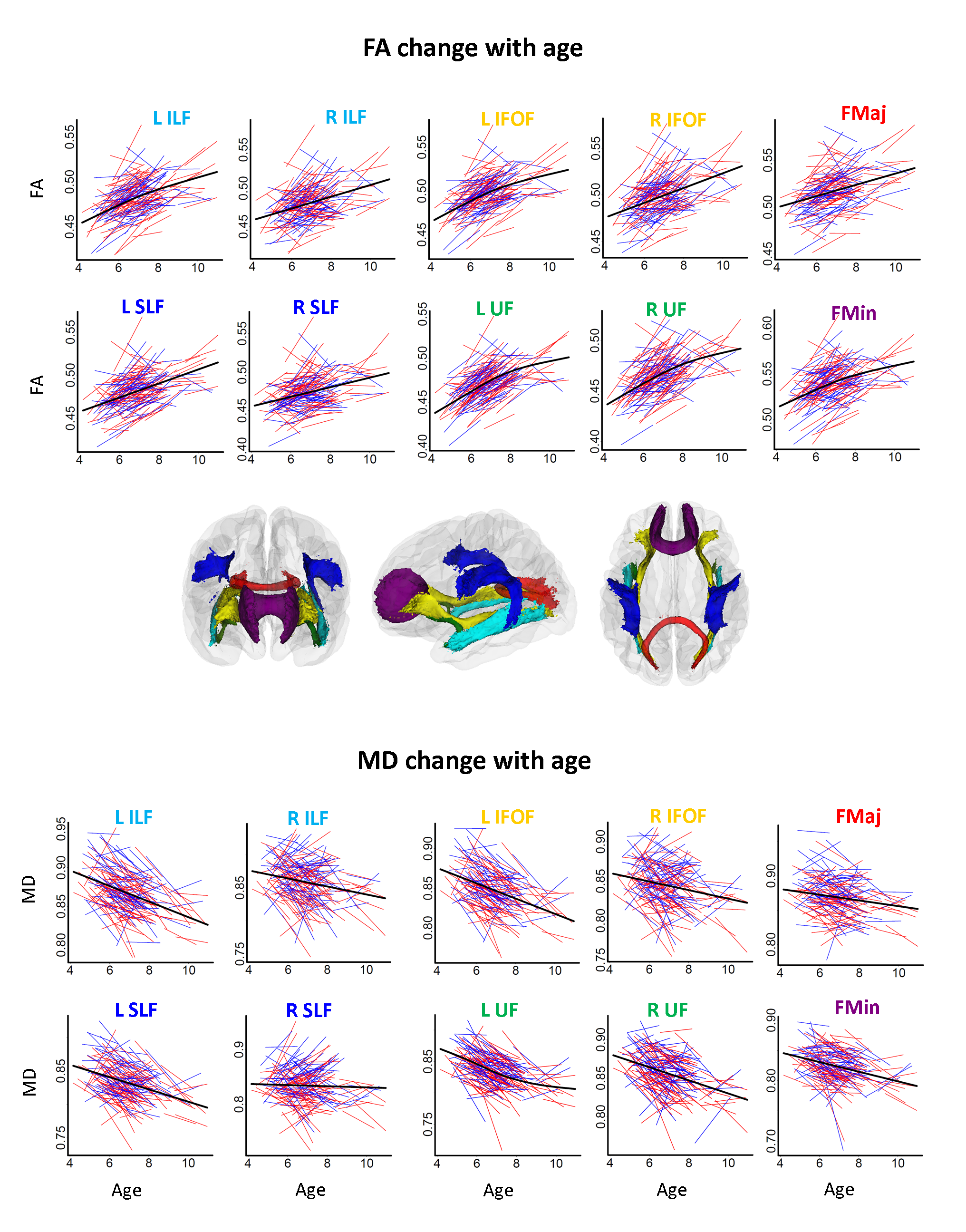

Supplement: S1 Fig — Spaghetti plots of individual participant change in FA and MD in specific tracts with age (years). Females are plotted in red and males in blue. For each measure, an assumption-free general additive mixed model as a function of age was fitted to accurately describe change across the age range. Three-dimensional renderings illustrate ten atlas-based probabilistic tracts from the Mori atlas in anterior, left, and dorsal views, displayed on a semitransparent template brain. The color-coded titles for each scatterplot represent the color of each specific white matter tract. Color codes refer to: Light blue: Inferior longitudinal fasciculus (ILF), Yellow: Inferior fronto-occipital fasciculus (IFOF), Red: Forceps major (FMaj), Blue: Superior longitudinal fasciculus (SLF), Green: Uncinate fasciculus (UF), and Purple: Forceps minor (FMin). The 3D figures were made by the use of Slicer (http://www.slicer.org/). L = left and R = right. (TIF) [file pone.0195540.s001.tif]

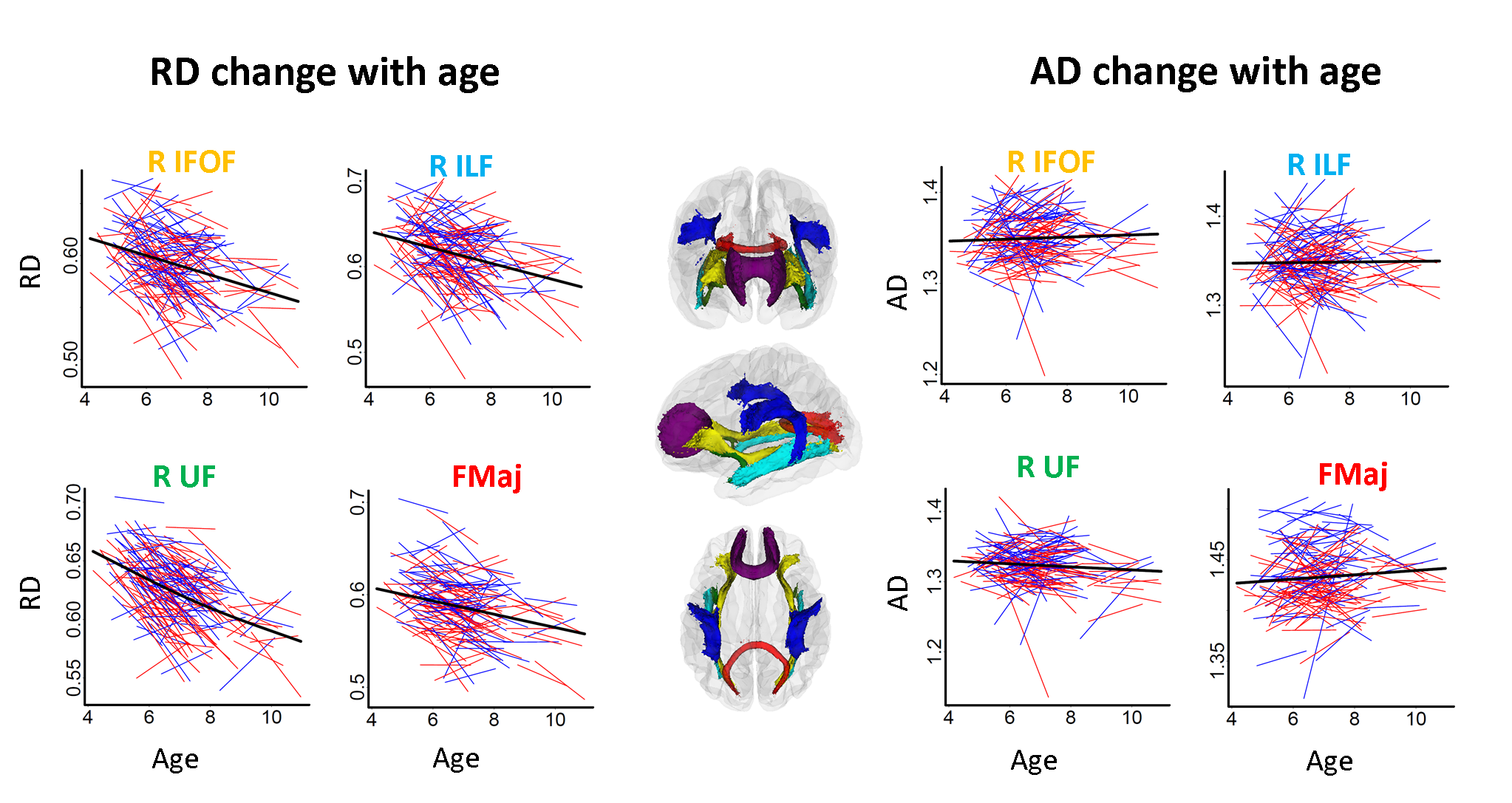

Supplement: S2 Fig — Spaghetti plots of individual participant change in RD and AD in specific tracts with age (years). Females are plotted in red and males in blue. For each measure, an assumption-free general additive mixed model as a function of age was fitted to accurately describe change across the age range. Three-dimensional renderings illustrate ten atlas-based probabilistic tracts from the Mori atlas in anterior, left, and dorsal views. The color-coded titles for each scatterplot represent the color of each specific white matter tract. Color codes refer to: Yellow: Inferior fronto-occipital fasciculus (IFOF), Light blue: Inferior longitudinal fasciculus (ILF), Green: Uncinate fasciculus (UF) and Red: Forceps major (FMaj). The 3D figures were made by the use of Slicer (http://www.slicer.org/). L = left and R = right. (TIF) [file pone.0195540.s002.tif]
