# Supplementary material for: Development of white matter microstructure in relation to verbal and visuospatial working memory—A longitudinal study
Source: PLoS One. 2018 Apr 24;13(4):e0195540. doi: 10.1371/journal.pone.0195540 (PMC5916522; doi:10.1371/journal.pone.0195540)
Supplement: S2 Table — Partial correlations between change in FA, MD, RD and AD in specific white matter tracts and change in Spatial Span Backward scores, controlling for age, sex, interval and motion at both time points after excluding three participants with SDR exceeding ±3 in some TOIs for FA, MD, RD and AD predicted by age. ILF = Inferior longitudinal fasciculus, IFOF = Inferior fronto-occipital fasciculus, UF = Uncinate fasciculus and FMaj = Forceps major. Numbers in bold signify Bonferroni-corrected significance level p < .031/.025/.027/.022 for FA, MD, RD and AD, respectively. (DOCX) [file pone.0195540.s004.docx]

**S2 Table. DTI change in white matter tracts and visuospatial working memory change after excluding outliers (for each TOI)**

|  |  |  | Spatial Span Backward | |
| --- | --- | --- | --- | --- |
| DTI metric | Tract | Hemisphere | r | p |
| FA | IFOF | right | .17 | .046 |
|  | FMaj |  | .10 | .229 |
|  | ILF | right | **-.20** | **.021** |
| MD | IFOF | right | **-.19** | **.022** |
|  | UF | right | -.16 | .055 |
|  | FMaj |  | -.19 | .026 |
|  | ILF | right | -.16 | .066 |
| RD | IFOF | right | -.17 | .041 |
|  | UF | right | -.16 | .062 |
|  | FMaj |  | -.17 | .050 |
|  | ILF | right | **-.20** | **.016** |
| AD | IFOF | right | -.17 | .047 |
|  | UF | right | -.10 | .264 |
|  | FMaj |  | -.16 | .057 |

Partial correlations between change in FA, MD, RD and AD in specific white matter tracts and change in Spatial Span Backward scores, controlling for age, sex, interval and motion at both time points after excluding three participants with SDR exceeding ±3 in some TOIs for FA, MD, RD and AD predicted by age. ILF = Inferior longitudinal fasciculus, IFOF = Inferior fronto-occipital fasciculus, UF = Uncinate fasciculus and FMaj = Forceps major. Numbers in bold signify Bonferroni-corrected significance level p < .031/.025/.027/.022 for FA, MD, RD and AD, respectively.
