# Supplementary material for: Development of white matter microstructure in relation to verbal and visuospatial working memory—A longitudinal study
Source: PLoS One. 2018 Apr 24;13(4):e0195540. doi: 10.1371/journal.pone.0195540 (PMC5916522; doi:10.1371/journal.pone.0195540)
Supplement: S6 Table — Partial correlation between change in AD in specific white matter tracts and change in Spatial Span Backward scores, controlling for age, sex, interval, motion at both time points and mean AD change. ILF = Inferior longitudinal fasciculus, IFOF = Inferior fronto-occipital fasciculus, UF = Uncinate fasciculus and FMaj = Forceps major. Numbers in bold signify Bonferroni-corrected significance level p < .05. (DOCX) [file pone.0195540.s008.docx]

**S6 Table. AD change in white matter tracts and working memory change, controlling for mean AD change**

|  |  | Spatial Span Backward | | Digit Span Backward | |
| --- | --- | --- | --- | --- | --- |
| Tract | Hemisphere | r | p | r | p |
| ILF | right | **-.28** | **<.001** | **-.18** | **.033** |
| IFOF | right | **-.26** | **.002** | -.09 | .316 |
| UF | right | -.12 | .146 | -.07 | .395 |
| FMaj |  | **-.22** | **.009** | -.03 | .691 |

Partial correlation between change in AD in specific white matter tracts and change in Spatial Span Backward scores, controlling for age, sex, interval, motion at both time points and mean AD change. ILF = Inferior longitudinal fasciculus, IFOF = Inferior fronto-occipital fasciculus, UF = Uncinate fasciculus and FMaj = Forceps major. Numbers in bold signify Bonferroni-corrected significance level p < .05.
