# Supplementary material for: Development of white matter microstructure in relation to verbal and visuospatial working memory—A longitudinal study
Source: PLoS One. 2018 Apr 24;13(4):e0195540. doi: 10.1371/journal.pone.0195540 (PMC5916522; doi:10.1371/journal.pone.0195540)
Supplement: S7 Table — Partial correlation between change in MD in specific white matter tracts and change in Spatial Span Backward and Digits Span Backward scores, controlling for age, sex, interval, motion at both time points and software upgrade. ILF = Inferior longitudinal fasciculus, IFOF = Inferior fronto-occipital fasciculus, SLF = Superior longitudinal fasciculus, UF = Uncinate fasciculus, FMaj = Forceps major and FMin = Forceps minor. Numbers in bold signify Bonferroni-corrected significance level p < .025. (DOCX) [file pone.0195540.s009.docx]

**S7 Table. MD change in white matter tracts and working memory change, controlling for software update**

|  |  | Spatial Span Backward | | Digit Span Backward | |
| --- | --- | --- | --- | --- | --- |
| Tract | Hemisphere | r | p | r | p |
| ILF | left | -.11 | .188 | .04 | .917 |
|  | right | **-.22** | **.008** | .02 | .934 |
| IFOF | left | -.13 | .124 | .07 | .674 |
|  | right | **-.23** | **.007** | .02 | .828 |
| SLF | left | -.06 | .460 | .09 | .424 |
|  | right | -.12 | .155 | .03 | .622 |
| UF | left | -.11 | .194 | .10 | .565 |
|  | right | **-.21** | **.013** | .01 | .896 |
| FMaj | | **-.24** | **.004** | -.03 | .834 |
| FMin | | -.16 | .052 | .10 | .429 |

Partial correlation between change in MD in specific white matter tracts and change in Spatial Span Backward and Digits Span Backward scores, controlling for age, sex, interval, motion at both time points and software upgrade. ILF = Inferior longitudinal fasciculus, IFOF = Inferior fronto-occipital fasciculus, SLF = Superior longitudinal fasciculus, UF = Uncinate fasciculus, FMaj = Forceps major and FMin = Forceps minor. Numbers in bold signify Bonferroni-corrected significance level p < .025.
