# Supplementary material for: Development of white matter microstructure in relation to verbal and visuospatial working memory—A longitudinal study
Source: PLoS One. 2018 Apr 24;13(4):e0195540. doi: 10.1371/journal.pone.0195540 (PMC5916522; doi:10.1371/journal.pone.0195540)
Supplement: S8 Table — Partial correlations between change in FA and MD in specific white matter tracts showing non-linear developmental patterns and change in Spatial Span Backward scores, controlling for age, sex, interval, motion at both time points and age2. ILF = Inferior longitudinal fasciculus, IFOF = Inferior fronto-occipital fasciculus, UF = Uncinate fasciculus and FMin = Forceps minor. (DOCX) [file pone.0195540.s010.docx]

**S8 Table. DTI change in non-linear white matter tracts and visuospatial working memory change, controlling for age^2^**

|  |  |  | Spatial Span Backward | |
| --- | --- | --- | --- | --- |
| DTI metric | Tract | Hemisphere | r | p |
|  | ILF | left | .07 | .416 |
| FA | IFOF | left | .07 | .418 |
|  | UF | left | .07 | .406 |
|  | UF | right | .12 | .143 |
|  | FMin |  | .15 | .087 |
| MD | UF | left | -.08 | .375 |

Partial correlations between change in FA and MD in specific white matter tracts showing non-linear developmental patterns and change in Spatial Span Backward scores, controlling for age, sex, interval, motion at both time points and age^2^. ILF = Inferior longitudinal fasciculus, IFOF = Inferior fronto-occipital fasciculus, UF = Uncinate fasciculus and FMin = Forceps minor.
